# Idiopathic Intestinal Angioedema: A Diagnostic Dilemma

**DOI:** 10.7759/cureus.4951

**Published:** 2019-06-20

**Authors:** Laith Numan, Suguni Loku Galappaththy, Nedaa M Husainat, Mouhanna Abu Ghanimeh

**Affiliations:** 1 Internal Medicine, University of Missouri-Kansas City School of Medicine, Kansas City, USA; 2 Kidney Institute, University of Kansas Hospital & Medical Center, Kansas City, USA; 3 Gastroenterology, Henry Ford Health System, Detroit, USA

**Keywords:** idiopathic, angioedema, intestine, allergy hypersensitivity, abdominal pain, omalizumab

## Abstract

Angioedema is an allergic reaction that usually involves the face and pharynx. Intestinal angioedema is a rare subtype that is typically linked to the use of angiotensin-converting enzymes inhibitors (ACEIs). Intestinal angioedema is challenging to diagnose, as it can mimic gastroenteritis or other inflammatory bowel conditions. Herein, we present a 34-year-old female who presented with recurrent episodes of abdominal pain. She underwent extensive workup for her abdominal pain and rash, and all was unrevealing except for high Immunoglobulin E (Ig E). Multiple imaging came back negative for any pathology. The allergy and immunology team evaluated the patient, and they believed her symptoms are likely caused by isolated intestinal angioedema with a histamine-related rash. She was started on high doses of antihistamines; her symptoms partially improved. Subsequently, she was started on a trial of omalizumab, which resulted in complete resolution of her symptoms. In conclusion, intestinal angioedema is a rare disease that should be suspected in cases of recurrent abdominal pain with negative workup, especially if the patient is taking ACEIs. Few cases were reported in the literature for patients on ACEI. In our case, the diagnosis was a challenge, as the patient was never on ACEI.

## Introduction

Angioedema encompasses a collection of syndromes that pose a significant diagnostic challenge to the clinician. The term angioedema describes a rapid, transient, localized swelling of the deeper layers of the skin. It is a result of the extravasation of fluid into interstitial tissue and typically affects areas with loose connective tissue such as the face, lips, mouth, throat, larynx, and gastrointestinal tract. The pathophysiology behind it is a consequence of increased vascular permeability caused by the release of vasoactive mediators. The mechanism can be either histamine-mediated or bradykinin-mediated, and with idiopathic cases, the mechanism is not fully understood. Given that 10%-20% of people worldwide will develop an episode of angioedema or urticaria at some point in their lifetime [1], it is essential to be aware of different presentations of angioedema.

Abdominal pain is a common presenting complaint in clinical practice, yet poses a diagnostic dilemma given the vast number of etiologies. Abdominal pain when presenting as the predominant symptom of idiopathic angioedema can be a challenging diagnosis to make, as it tends to overlap with other similar conditions. Furthermore, once the diagnosis of idiopathic angioedema is made, the management continues to remain complicated, as there is unpredictable response and it is typically recalcitrant to available treatment [1]. This has been demonstrated by multiple past case studies [2].

Herein, we present a case presenting with recurrent episodes of abdominal pain and rash, which was diagnosed with idiopathic angioedema and was not responsive to first-line treatment.

## Case presentation

A 34-year-old Caucasian female with a history of aspirin-exacerbated respiratory disease (AERD) presented with recurrent episodes of diffuse colicky abdominal pain. She also has an urticarial rash, flushing, and profuse sweating. Bouts of non-bloody, watery diarrhea accompanied the abdominal pain. No identifiable trigger was identified, and there was no association with a specific food type allergy. She denied any weight loss. On presentation, she was afebrile, normotensive, and slightly tachycardic. Her abdomen was tender to palpation. Her pelvic exam was normal.

She has had a similar episode in the past; the most recent being three months prior, which was also associated with an itchy rash and abdominal pain and diarrhea. She also has had multiple visits to the Emergency Department (ED), including on the previous admission. She had undergone a comprehensive list of investigations before her presentation at our institution. Lab workup was done to exclude viral exanthems, including Epstein-Bar Virus (EBV) and Cytomegalovirus (CMV) as well as an autoimmune antibodies panel; all which came back negative. Further workup included a Computed Tomography (CT) scan of the abdomen and pelvis with contrast and a Hepatobiliary Iminodiacetic Acid (HIDA) scan; all which showed no significant abdominal or gynecological findings. Given the episodic nature of her symptoms, serum metanephrines and normetanephrines were also tested and were negative. Gastroenterology was consulted, and she underwent an Esophagogastroduodenoscopy (EGD)-guided biopsy; biopsy revealed mild gastritis. Her drug history included a budesonide inhaler for her asthma and metformin for he Polycystic Ovarian Syndrome (PCOS) as well as Diphenhydramine; to which the patient reports that it helps relieve some of her current symptoms. She denies using oral contraceptives or an Angiotensin Converting Enzyme Inhibitor (ACE-I).

Considering the patients' extensive history and work-up, our investigations were focused on developing a set of differentials that present with a recurrent generalized abdominal and a progressively worsening rash. Initial bloodwork was significant for leukopenia and elevated C-Reactive Protein (CRP). She had a negative Hepatitis Panel, Normal Thyroid Stimulating (TSH) hormone, and repeat CT and EGD with subsequent colonoscopy showed no significant pathology. The 24-hour urine 5-Hydroxyindoleacetic acid (5-HIAA) levels and Tryptase levels were also negative. Skin punch biopsy with histological evaluation using direct immunofluorescence revealed weak Immunoglobulin A (IgA) deposition of underdetermined sequence. No vasculitis was identified, and findings were more indicative of urticaria. Serum Immunoglobin E (IgE) was elevated at 178 IU/ml (normal range: 0-100 IU/ml). C1 esterase inhibitor level was normal.

Rheumatology was consulted, and they recommended that the patient be started on prednisone 40 mg daily to be tapered off over six weeks for the treatment of possible Henoch-Schönlein purpura (HSP). However, this prednisone was tapered off after it showed no or minimal response in the patient's condition. Subsequently, the allergy and immunology team was consulted. Their assessment discerned that the patient lacked classical features of hereditary angioedema. Despite the inadequate response to steroids, the patient did report some symptomatic relief with Diphenhydramine. This concludes that the rash likely has a histamine-mediated component rather than the bradykinin-associated angioedema associated with transient intestinal angioedema. The patient was started on a trial of high dose Cetirizine 20 mg twice daily. After a few days of inadequate response with this treatment, Ranitidine 300 mg daily and Montelukast 10 mg daily were added to this regimen. This treatment continued for several weeks with an inadequate response before it was decided to attempt a trial of Omalizumab. After a month of treatment with the Omalizumab, the patient was finally symptom-free. On her six months follow-up, the patient reported complete resolution of her abdominal pain and rash and had no further episodes since discharge.

## Discussion

Idiopathic intestinal angioedema, as the name suggests, is essentially a diagnosis of exclusion. The terms idiopathic recurrent angioedema is used when three or more episodes of angioedema have occurred within a period of six months to one year without a cause being identified after a comprehensive medical evaluation. Similar to our patient's presentation, 50% of cases of angioedema are accompanied by urticaria and pruritis [3]. It must be emphasized that the successful management of these patients requires a multidisciplinary approach that will likely involve multiple services, including an internist, gastroenterologist, rheumatologist, and immunologist, before a final diagnosis can be reached.

The initial evaluation should comprise a detailed personal and family history, including identification of triggers of angioedema such as food allergens, latex allergies, and insect bites. Medications also constitute an essential portion of this history, especially ACE-Is and Non-Steroidal Anti-inflammatory Drug (NSAIDs), which are well-known causes of angioedema. Of note, in patients such as ours with a known history of AERD, there is a common variant of AERD that has predominantly dermatological and abdominal symptoms, which would present as abdominal pain, nausea, and vomiting, with an erythematous pruritic macular rash on the extremities and palmar surfaces, which is not accompanied by urticaria or angioedema [4]. However, our patient has been off aspirin and other NSAIDs since the diagnosis of Samter’s triad several years back. Therefore, the likelihood of this being the cause is unlikely.

Angioedema, as a physical finding, may affect any part of the gastrointestinal symptom and is usually more distressing to most patients than dermatological manifestations. Visceral edema in the digestive system can manifest as nausea, vomiting, abdominal pain, diarrhea, and constipation, depending on the location that is involved. The most common manifestation of gastrointestinal angioedema is abdominal pain, which can vary in severity and has been described as severe to excruciating in 87% of patients [5]. Other accompanying gastrointestinal manifestations may include vomiting and diarrhea, which can occur in 78% and 65% respectively in patients who present with coexisting abdominal pain [5]. These symptoms are considered a manifestation of bowel wall edema, which is a transient and completely reversible condition.

The initial workup generally includes complete blood count, comprehensive metabolic panel, protein electrophoresis, total serum IgE, erythrocyte sedimentation rate (ESR), complement levels, and thyroid cascade [1]. Thyroid cascade and antithyroid autoantibodies are an essential consideration, as several studies have linked microsomal and antithyroglobulin autoantibodies associated with Hashimoto's thyroiditis with recurrent angioedema and urticaria [6-7]. C1 esterase is inhibitor levels and functional levels are also recommended to evaluate recurrent angioedema [1], but in our patient who also had urticaria, Hereditary Angioedema (HAE) with C1 inhibitor deficiency (which does not present with urticaria) was not in the differential diagnosis.

Radiologic studies are usually a useful modality of the investigation but have not been proven to be definitive in the diagnosis of this condition. Contrast-enhanced CT scans often demonstrate findings that include intestinal wall and mucosal thickening, which is consistent with bowel edema and ascites [8]. X-ray findings may reveal various degrees of bowel obstruction with or without fluid levels. These discoveries are more commonly evident during an acute presentation and are often not seen in between episodes. At this point, another pertinent point to bring up would be that mild ascites and bowel wall thickening may commonly be overlooked by a radiologist, particularly in cases with low suspicion of intestinal angioedema [9]. Therefore, it would be essential to maintain that a negative finding on radiological examination may not exclude this diagnosis, as illustrated in the case of our patient. Further, this should not preclude the clinician from pursuing further imaging and investigations during a subsequent attack.

Idiopathic angioedema is a challenging condition to treat. Generally, corticosteroids and H-1 antihistamines are the first-line agents for treatment. In some cases, H2 receptor agonists are added to the treatment plan to aid in the inhibition of a histamine-induced rash. In our case, the patient was not responding to steroids and displayed an incomplete response to antihistamines. This finding is reinforced by several studies that have revealed that even in cases of chronic idiopathic/spontaneous urticaria (CIU/CSU), complete symptomatic relief is observed in <50% of the cases [10]. This is even more poignant in the cases of urticaria-associated angioedema. In one study, it was shown that angioedema was more common in patients with CIU/CSU unresponsive to treatment as compared with patients responsive to treatment (59% vs. 29%) [11]. This further illustrates the unknown nature of idiopathic angioedema and the difficulty in finding targeted pharmacologic treatments for its management.

On the prospect of emerging management modalities, there are a few pharmacological agents that are now being looked at as possible treatment options for idiopathic angioedema. Omalizumab is a recombinant humanized monoclonal anti-IgE antibody that is Food and Drug Administration (FDA) approved to treat asthma and urticaria. There have now been several cases reported of idiopathic angioedema that have been treated with Omalizumab when the condition has been resistant to treatment with first-line therapies [12-13]. This treatment strategy hypothesizes that the IgE pathway mediates a subset of Idiopathic angioedema despite our inability to find an IgE-related trigger in our clinical investigations. In our patient, this finding may be supported by the mild elevation in IgE noted on the patient's serology.

This case was presented as a poster at the American College of Gastroenterology annual meeting (Numan L, et al. Idiopathic Intestinal Angioedema: A Diagnostic Dilemma. ACG 2018 Annual Scientific Meeting Abstracts. Philadelphia, Pennsylvania).

## Conclusions

Idiopathic angioedema continues to be a diagnostic dilemma for most practitioners. As illustrated by our case, the illness continues to manifest with variable presentations and clinical courses. As our understanding of the condition progresses further, the scientific community believes that more innovative management strategies are forthcoming, both in the diagnosis and in the management of this disease.
